# Doctors’ Perception Regarding Bariatric Surgery and Major Barriers in Referral of Morbidly Obese Patients for Surgery in Khyber Pakhtunkhwa

**DOI:** 10.7759/cureus.40305

**Published:** 2023-06-12

**Authors:** Mohammad Zarin, Zia Ullah, Muhammad I Khan, Shahzeb Khan, Syed Asad Maroof, Mutahar Bashir

**Affiliations:** 1 General Surgery, Khyber Medical College/Khyber Teaching Hospital, Peshawar, PAK; 2 Health and Nutrition Program, Helping Hand for Relief and Development, Islamabad, PAK

**Keywords:** weight-loss, management of obesity, bariatric surgery, morbid obesity, body mass index

## Abstract

Introduction: Obesity is on the rise worldwide and has emerged as a global health concern. It has presented itself as the leading cause of morbidity, disability, and healthcare utilization. Bariatric surgery is a viable treatment option that offers sustained weight loss and improvement in comorbidities. The aim of this study is to determine the perception of doctors regarding bariatric surgery and the major barriers to the referral of morbidly obese for surgery.

Method: This study is a cross-sectional descriptive study conducted from November 1, 2022, to December 31, 2022. It involved prospective data collection through online questionnaires filled by doctors practicing in Peshawar. The sampling technique was non-probability convenience-based sampling. The sample size was 152. Doctors from all age groups and both genders were included in our study. Non-consenting doctors and those who were practicing bariatric surgery were excluded. Data were analyzed using a statistical package for social sciences (SPSS) version 25.0 (IBM Inc., Armonk, NY). Categorical variables have been presented as frequencies and percentages. Numerical variables have been presented as mean ± SD.

Results: A total of 152 doctors participated in our research study; 92 were physicians and 60 were surgeons. The majority of our study participants' patient load per week was >75. Around 47% believed bariatric surgery was a valuable tool in the treatment of morbid obesity. The most commonly reported barrier to referral was surgical complications or side effects (28.9%).

Conclusion: The study concluded that the awareness regarding bariatric and metabolic surgery remains flimsy among the doctor community. Most of the physicians were unaware of the benefits of the surgical management of obesity. They also had doubts regarding the safety of the procedure. We need proper utilization of awareness strategies to overcome these barriers.

## Introduction

For the measurement of obesity, body mass index (BMI) is used as a standard gauge. Every person with a BMI of 25-29.99 kg/m^2^ is considered overweight, while those having BMI above 30 kg/m^2^ are considered obese. The World Health Organization (WHO) defines Class III obesity as having a BMI above or equal to 40 kg/m^2^ [1]. Obesity is a predisposing factor for several comorbidities including diabetes type 2, obstructive sleep apnea, cardiovascular diseases, infertility in women, several types of cancers, and depression [2]. Obesity is a known cause of higher mortality, poorer quality of life, and higher financial burden on the healthcare system and society due to higher rates of absenteeism, productivity loss, and premature mortality [3].

According to the WHO, more than 340 million people aged 5-19 are overweight or obese worldwide [4]. Data from the U.S. National Centre for Health Statistics show that 35% of adults in the United States are obese [5]. Obesity is ranked as the second-leading cause of preventable deaths in the United States; it is a leading cause of morbidity, disability, and healthcare utilization and increases healthcare costs [6]. According to the data from the American Society for Metabolic and Bariatric Surgery, in 2011-2017, <1% of the American population who were eligible for bariatric surgery underwent the said surgery for morbid obesity [7]. The number of bariatric procedures in the United States has increased remarkably over the last decade with approximately over 200,000 surgeries being performed annually [8].

There are many reasons for the strikingly low inclination toward bariatric surgery [9]. Wee et al. described the relationship between healthcare providers’ recommendation and patients’ inclination to choose a bariatric surgical procedure for their obesity-related comorbidity, showing that patients were five times more likely to choose such a procedure if their healthcare provider suggested it than those whose doctors did not. These researchers also found that doctors’ recommendation was a stronger predictor than patient gender, age, race, BMI, or other obesity-related health problems, which further enforced the role of primary physicians in the utilization of bariatric surgery as an option to treat morbid obesity [10].

Little Indigenous study is being done on the topic, and hence few data are gathered from local healthcare centers. This study was designed to assess doctors’ knowledge and perceptions regarding bariatric surgery in local doctors of Khyber Pakhtunkhwa (KP). It was also aimed at clarifying their concerns about patients’ referrals for bariatric surgery.

## Materials and methods

This study is a cross-sectional study. The study population included doctors working in different hospitals in KP. The study was conducted from November 1, 2022, to December 31, 2022. The research proposal was presented to and was approved by the Institutional Research and Ethical Review Board (IREB KMC).

To assess the perception and referral barriers among the study population, a pretested questionnaire was used. The questionnaire was self-administered. Both online and hardcopy submissions were entertained. The online copy of the survey was developed using Google Forms. Verbal consent was sought from all the participants with assurance of complete confidentiality. Non-disclosure of information was promised. We calculated the sample size with OpenEpi. The confidence level was 90%, the margin error was 10%, and the power was 50%. Our estimated sample size was n = 112. We used consecutive sampling for convenience. The study population included doctors practicing in medicine and allied as well as surgery and allied fields. Consultants, fellows, and registrars were included. Doctors practicing bariatric surgery and non-consenting doctors were excluded from the surgery. Data were stored and analyzed using SPSS version 25.0 (IBM Inc., Armonk, NY). Statistical analysis was done and measures of frequencies and percentages were documented.

All authors have read and approved the final manuscript. 

## Results

A total of 152 doctors participated in our research study. Among them, 40 were female and 112 were male (Table 1). All of our study participants were consultants. Among them, 92 were physicians and 60 were surgeons (Table 2). The majority of our study participants’ patient load per week was >75 patients (56.6%). 

**Table 1:** Gender distribution

Gender	Frequency	Percentage
Female	40	26.3
Male	112	73.7
Total	152	

**Table 2:** Fields of consultants

Specialty	Frequency	Percentage
Physician	92	60.5
Surgeon	60	39.5
Total	152	

In reply to the declaration “The results of bariatric surgery are too good in the management of patients with morbid obesity,” the response from 47.4% of doctors was either agree or strongly agree, while 52.6% of doctors responded that they disagreed or strongly disagreed (Table 3).

**Table 3:** Perception of consultants The results of bariatric surgery are too good in the management of patients with morbid obesity.

Response	Frequency	Percentage
Agree	50	32.9
Strongly Agree	22	14.5
Disagree	44	28.9
Strongly disagree	36	23.7
Total	152	

In our study, 80.2% of the physicians had some barriers to referring an obese patient to a bariatric surgery center. In our study, 38.1% of the doctors identified surgical complications and ineffectiveness of the bariatric procedures as barriers to referring a patient to bariatric surgery. The two highest-reported barriers to referral were surgical complications or side effects (28.9%) and lacking familiarity with available options of bariatric surgery (22.4%). For 17.1% of our respondents, socioeconomic factors were the reported barriers to referring a patient (Table 4).

**Table 4:** Referral barriers

Barrier	Frequency	Percentage
Surgical complications or side effects	44	28.9
Lack of familiarity with bariatric surgical options	34	22.4
Socioeconomic factor	26	17.1
Postoperative long-term care	16	10.5
Ineffective postoperative weight loss	14	9.2
No barriers to referral	12	7.9
Other	6	3.9
Total	152	

**Figure 1:**
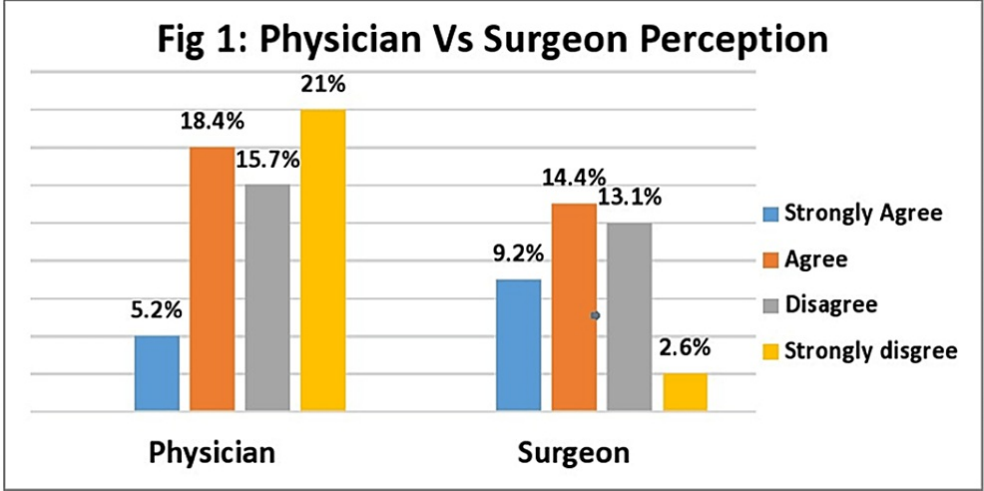
Physicians vs surgeons: Perception regarding effectiveness of bariatric surgery

There was a significant difference in the perception of physicians and surgeons regarding the effectiveness of bariatric surgery (Figure 1).

## Discussion

Bariatric surgery has established itself as a viable treatment option for the management of morbid obesity. An obese patient typically first presents to his/her primary care physician. The doctor and the patient considering bariatric surgery as an option is the first step toward a favorable outcome. Memarian et al. found that in Sweden, most bariatric surgery patients are referred by their primary care physicians. In the majority (84%) of these cases, the idea to pursue bariatric surgery is initiated by the patients. Similar to our study, they found that half of the physicians considered bariatric surgery as favorable for their patients. In our study, 47.7% of the doctors agreed that bariatric surgery is an important option in the treatment of morbid obesity. Bariatric surgery has been proven to decrease BMI effectively and thus the comorbidities of obesity. These data indicate a lack of awareness among physicians about the updated data on bariatric surgery and its results. Various means could be utilized to increase access to the ever-evolving literature about the advancements and usefulness of bariatric surgery. Almost half of the doctor community, both here and abroad, have some reservations about the guidelines on how to treat morbid obesity. In Memarian et al.’s study, 44% of the physicians agreed that they needed to educate themselves and learn more about bariatric surgery, whereas around 13.2% of our respondents answered that they were deficient in their knowledge about the currently available options for bariatric surgery [11].

In our study, doctors identified surgical complications and lack of familiarity with bariatric surgical options as the major hurdles in referring a patient to bariatric surgery. They also found that patients were more likely to undergo bariatric surgery if they were advised by their primary care physicians to consider it. In our study, it can be interpreted that there was a lack of adequate information as to how useful bariatric surgery is to the target population, which led to fewer patient referrals from the physicians for bariatric surgery. A national awareness campaign on this subject is needed, whereby both doctors and the general population may gain a basic understanding [7]. A study conducted in Pakistan showed that 22.8% (2,296 out of 10,063) of adults were overweight and 5.1% (512 out of 10,063) were obese [12]. The majority of obese people develop some sequelae of obesity and ultimately utilize some kind of healthcare resources. In a review of the cost-effectiveness of bariatric surgery, Wang et al. found that bariatric surgery is the most cost-effective treatment of obesity in the long term. Proper and timely guidance of these patients and their primary care physicians can lead to enhanced referrals for bariatric procedures. This will eventually decrease the healthcare burden while also improving the patients’ well-being and quality of life [13].

In a study conducted in the United States, Ouni et al. found that 52.3% of primary care physicians were unaware that bariatric surgery was an intervention for weight loss and BMI control. Although 46.2% of the physicians in that study agreed that bariatric surgery is an effective modality for weight loss, only 24.6% of participants were familiar with the criterion of referral for bariatric surgery. Of the participants, 53.1% had experienced one barrier or another to referring their patients for bariatric surgery. A similar picture can be seen in our results, whereby only 47.7% of the physicians identified bariatric surgery as an effective treatment for obesity, and the other half of the participants disagreed. In our study, 80.2% of the physicians found some barriers to referring an obese patient to a bariatric surgery center. Adding to that, 13.2% of the doctors found that after their recommendation, the patients were reluctant about the idea of bariatric surgery for obesity. This reflects that a large number of our doctors and patients are reticent regarding bariatric surgery and need to be provided with updated information [14].

Fewer than 1% of obese patients in the United States who fulfilled the criterion underwent bariatric surgery to treat obesity and obesity-related comorbidities [7]. The same statistics were found over a period of six years from 2011 to 2017. Our study suggests that a lack of knowledge among primary care physicians about the safety and efficacy of bariatric surgery greatly affects the referral of eligible patients to a bariatric surgeon. In a similar study, 21.5% of participants identified surgical complications as a main barrier to referral, and 18.5% expressed concern regarding the ineffectiveness of the bariatric procedures resulting in significant weight loss. Combined, this equals 40% of the study participants [15]. In our study, 38.1% of the participants expressed the same concerns. A general similarity of perception is palpable among doctors locally and internationally. Studies have shown that physicians are widely found to be reluctant to refer patients to bariatric surgery for weight loss [15].

## Conclusions

By and large, awareness regarding bariatric and metabolic surgery remains insubstantial among the doctor community. For most, it is still an unfamiliar specialty. This is despite its proven efficacy in facilitating sustained weight loss and combating several obesity-related comorbidities. As well as being a dilemma for bariatric surgeons, this also negatively impacts the health problems associated with it in the general population. A conscious, focused effort needs to be made to educate healthcare providers about the evolution of bariatric procedures, the potential benefits they offer, and the existence of certified bariatric centers. This will allow doctors to guide their patients toward optimum health care and enable them to benefit from bariatric surgery. Besides, doctors have hinted toward certain barriers that hinder their referral of patients for metabolic surgeries. There is a need to implement awareness strategies to overcome these barriers, specifically the commonly known surgical complications and weight loss outcomes.
